# The Dynamics of *Mycoplasma gallisepticum* Nucleoid Structure at the Exponential and Stationary Growth Phases

**DOI:** 10.3389/fmicb.2021.753760

**Published:** 2021-11-18

**Authors:** Gleb Y. Fisunov, Alexander I. Zubov, Olga V. Pobeguts, Anna M. Varizhuk, Mariya A. Galyamina, Daria V. Evsyutina, Tatiana A. Semashko, Valentin A. Manuvera, Sergey I. Kovalchuk, Rustam K. Ziganshin, Nicolay A. Barinov, Dmitry V. Klinov, Vadim M. Govorun

**Affiliations:** ^1^Federal Research and Clinical Center of Physical-Chemical Medicine of Federal Medical Biological Agency, Moscow, Russia; ^2^Shemyakin-Ovchinnikov Institute of Bioorganic Chemistry, Russian Academy of Sciences, Moscow, Russia

**Keywords:** *Mycoplasma gallisepticum*, nucleoid, nucleoid-associated proteins, enolase, proteome

## Abstract

The structure and dynamics of bacterial nucleoids play important roles in regulating gene expression. Bacteria of class Mollicutes and, in particular, mycoplasmas feature extremely reduced genomes. They lack multiple structural proteins of the nucleoid, as well as regulators of gene expression. We studied the organization of *Mycoplasma gallisepticum* nucleoids in the stationary and exponential growth phases at the structural and protein levels. The growth phase transition results in the structural reorganization of *M. gallisepticum* nucleoid. In particular, it undergoes condensation and changes in the protein content. The observed changes corroborate with the previously identified global rearrangement of the transcriptional landscape in this bacterium during the growth phase transition. In addition, we identified that the glycolytic enzyme enolase functions as a nucleoid structural protein in this bacterium. It is capable of non-specific DNA binding and can form fibril-like complexes with DNA.

## Introduction

Bacterial nucleoid structure and function are maintained by a set of proteins, including structural proteins that organize DNA loops, proteins that maintain negative supercoiling of DNA, RNA polymerase (RNAP), transcription factors and proteins that facilitate nucleoid separation into daughter cells during cell division. The nucleoid structure and its dynamics represent an important part of global gene expression regulation. Nucleoid-associated proteins (NAPs) can feature recognition preferences for sequences, local DNA structures and topologies (Duzdevich et al., 2014). Nucleoid-associated proteins may form bridges between distant DNA loci or topologically isolated DNA domains, modulating the local degree of supercoiling or accessibility of RNAP to promoters (Macvanin and Adhya, 2012; Song and Loparo, 2015; Lioy et al., 2018). The integration of the known data for regulatory networks in *Escherichia coli* demonstrates that nucleoid structural proteins may serve as high-level nodes in regulatory networks (Martínez-Antonio and Collado-Vides, 2003).

The H-NS protein, which is among the major nucleoid structural proteins, prefers AT-rich sequences. The binding of H-NS to AT-rich genomic regions results in the repression of 5–10% of the genes in *E. coli* (Gordon et al., 2011). The regulation of the 16S rRNA gene *rrnB* in *E. coli* occurs through the isolation of the gene promoter within the chromosomal loop stabilized by H-NS (Dame et al., 2002). IHF and H-NS in pair function as positive and negative regulators of *phoU* gene transcription in *E. coli*, respectively (Khodr et al., 2015). In *Desulfovibrio vulgaris*, IHF functions as an activator of σ^54^-dependent promoters (Fiévet et al., 2014). It has been demonstrated that the HU protein can affect the compaction state of DNA on a global level in response to environmental changes, such as acid stress (Remesh et al., 2020). This, in turn, affects the global transcriptional landscape. In this case, the HU protein functions as both an effector and a sensor. The phenomenon of liquid-liquid phase separation (LLPS) of NAPs is another example of nucleoid structure dynamics that globally regulates gene expression. LLPS has been described for RNAP and NusA in *E. coli* (Ladouceur et al., 2020). This effect is dependent on the growth phase. The degree of genomic DNA supercoiling maintained by DNA gyrases and topoisomerases is an important regulator of gene expression. There are several mechanisms underlying supercoiling-dependent regulation. More negative supercoiling decreases the energy required for promoter opening. Promoters that are sensitive to the global degree of DNA supercoiling have been described for chlamydia (Niehus et al., 2008). Another mechanism involves the supercoiling-induced formation of cruciform DNA at the promoter region, which disrupts the promoter structure (Horwitz and Loeb, 1988). The degree of genomic DNA supercoiling is involved in the regulation of MG_149 in *Mycoplasma genitalium* under osmotic stress (Zhang and Baseman, 2011). Nucleoid structural proteins can preferentially bind to DNA with a certain degree of supercoiling or bending. The Fis protein of *E. coli* can maintain topological homeostasis, preventing an extreme shift of supercoiling (Schneider et al., 2001; Skoko et al., 2006; Cameron et al., 2011). The HU protein bends DNA upon binding, introducing local negative supercoiling (Wojtuszewski et al., 2001; Swinger et al., 2003; Guo and Adhya, 2007).

Bacteria of class Mollicutes in general and *Mycoplasma gallisepticum*, in particular, feature extremely reduced genomes as well as protein repertoires. The set of structural nucleoid proteins in *M. gallisepticum* was reduced to two homologs of the HU protein. One of them lacks non-specific DNA-binding activity and may represent a site-specific binder or have a function different from DNA binding (Kamashev et al., 2011). In addition, the group of transcription factors (TFs) or TF-resembling proteins comprises 5–10 proteins, depending on the degree of homology with known proteins (Fisunov et al., 2016b). The structure of *M. pneumoniae* nucleoids has been extensively studied using Hi-C and super-resolution microscopy (Trussart et al., 2017). The data obtained helped to reveal the structure of the chromosome-interacting domains with 10 kb resolution. Their size ranged from 15 to 33 kb and genes within the same domain showed a tendency to co-regulate. In the exponential phase, the probability of interaction between chromosomal loci appeared to be directly proportional to their proximity. The authors proposed that this could have happened because of population heterogeneity within the non-synchronized culture (Trussart et al., 2017).

Previously obtained transcriptomic data indicate that *M. gallisepticum* undergoes global rearrangement of the transcriptional landscape depending on the growth phase (Mazin et al., 2014). During the stationary phase transition, the majority of the genes undergo significant repression. At the same time, a minor fraction of genes is activated (Mazin et al., 2014). This process cannot be explained by the existing repertoire of specific regulators. To reveal the underlying mechanisms, we studied the structure and protein composition of the *M. gallisepticum* nucleoid during the exponential and stationary growth phases.

## Materials and Methods

### *Mycoplasma gallisepticum* Cultivation and Genetic Transformation

*Mycoplasma gallisepticum* strain *S6* was obtained from the collection of microorganisms at the N. F. Gamaleya National Research Center (Moscow, Russia). A culture of *M. gallisepticum* with the synchronized division was obtained according to a previously described method (Kelton, 1962). The culture of *M. gallisepticum S6* was grown in a liquid medium containing tryptose (20 g/L), Tris (3 g/L), NaCl (5 g/L), KCl (5 g/L), horse serum (10% Biolot, Russia), glucose 1% (Sigma) and penicillin (Sintez, Russia), with a final concentration of 500 units/mL, at pH 7.4 and 37°C. One percent of the mycoplasma culture grown to the stationary phase of growth was placed in a starved medium containing only tryptose (20 g/L), Tris (3 g/L), NaCl (5 g/L), KCl (5 g/L) and cultured for 9 h at 37°C. Next, 10% yeast extract, 20% horse serum and 1% glucose were added. The culture was grown at 37°C to the logarithmic or stationary growth phases. The synchronized cultures were used for nucleoid isolation at both growth phases.

For quantitative gene expression measurements, the previously designed transposon vector based on pBluescript SK + backbone (Mazin et al., 2014) was modified to carry the gene for mMaple2 reporter protein (Rumyantseva et al., 2019). The assembly of genetic constructs was performed in *E. coli* Top-10 strain. For this the vector carried ampicillin resistance gene. The assembled vectors with mMaple2 coding sequence, respective promoter and Shine-Dalgarno sequence were used for genetic transformation of *M. gallisepticum* via electroporation (Mazin et al., 2014). The mMaple2 gene was chemically synthesized according to the mycoplasma codon usage table. The DNA fragments carrying the promoters and Shine-Dalgarno sequences were chemically synthesized and ligated into the vector upstream of the mMaple2 gene. The strong promoter featured the EXT-element (TATG), consensus -10-box (TATAAT) and strong initiator nucleotide G. The weak promoter lacked the EXT-element, featured an alternative -10-box (TAAAAT) and a less efficient initiator nucleotide A. The minimal strong promoter consensus for *M. gallisepticum* is **TRTG**NTATAATN_6_^∗^R, where R = A or G, the EXT element is highlighted in bold, -10-box is underlined and ^∗^ indicates the position of the transcription start site (Mazin et al., 2014).

### Nucleoid Isolation

Nucleoid fractions were isolated using the method described by Murphy and Zimmerman (1997) with modifications. Cells (50 mL of culture for the logarithmic or stationary phase) were harvested using centrifugation at 10,000 × *g* at 4°C for 10 min and washed twice with cold wash buffer [50 mM Tris-HCl (pH 7.4), 150 mM NaCl and 3 mM MgCl_2_]. The cell pellets were resuspended in 0.5 mL of solution A containing 10% sucrose, 10 mM Tris-HCl (pH 8.2), 100 mM NaCl, 20% sucrose and protease inhibitor cocktail (GE HealthCare, United States). Then 0.5 ml of solution B [10 mM Tris-HCl (pH 8.2), 100 mM NaCl, 10 mM EDTA, 10 mM spermidine (Sigma-Aldrich, United States) and 2% NP-40 (Sigma-Aldrich, United States)] was added. After incubation for 10 min, the cell lysate was loaded onto a sucrose gradient (10 mL total gradient volume in 15 mL plastic tubes, using gradients with a linear increase from 20 to 60% sucrose in Solution A). The samples were then centrifuged for 90 min at 10000 × *g* at 4°C. The viscous clot in the middle of the sucrose gradient was removed from the tubes. Nucleoids were then washed with 10 mM Tris-HCl (pH 7.4) containing 100 mM NaCl and centrifuged for 10 min at 3000 × *g* at 4°C.

### Proteomic Analysis

#### Tryptic Digestion

Sample preparation for proteomic analysis was performed as follows: the samples were lysed in a lysis buffer containing 1% sodium deoxycholate (SDC, Sigma), 100 mM Tris-HCl (pH 8.5) with a protease inhibitor cocktail (GE Healthcare) through ultrasonication with a Branson 1510 sonicator at 4°C for 1 min, duty cycle 10%. Protein concentration was estimated using the BCA assay (Sigma). Aliquots containing 300 μg of the protein material were diluted to 1 μg/μL with lysis buffer and Tris (2-Carboxyethyl) phosphine hydrochloride (TCEP, Sigma) and chloroacetamide (CAA, Sigma) were added to final concentrations of 10 and 30 mM, respectively. Cys-reduction and alkylation were achieved by heating the sample for 10 min at 85°C. Trypsin (Promega, United States) was added at a ratio of 1:100 w/w to the protein amount and incubated at 37°C for overnight. Then, the second trypsin portion 1:100 w/w was added and the sample was incubated for 4 h at 37°C. Proteolysis was stopped by the addition of 1% trifluoroacetic acid. The precipitated SDC was removed using ethyl acetate (Masuda et al., 2008). The samples were purified using OASIS columns (Water) and analyzed using liquid chromatography-mass spectrometry (LC-MS).

#### Liquid Chromatography-Mass Spectrometry Analysis

Liquid chromatography-mass spectrometry (LC-MS) analysis was carried out on an Ultimate 3000 RSLCnano HPLC system connected to a Fusion Lumos mass spectrometer, controlled by XCalibur software version 4.3.73.11 (Thermo Fisher Scientific). Each sample was injected with a homemade iRT peptide mixture (Escher et al., 2012). The samples were loaded to a 20 × 0.1 mm PepMap C18 5 m trap column (Thermo Fisher Scientific) in loading buffer [2% acetonitrile (ACN), 98% H_2_O and 0.1% trifluoroacetic acid (TFA)] at 10 μL/min flow and separated at RT in a home-packed 300 × 0.1 mm fused-silica pulled emitter column packed with Reprosil PUR C18AQ 1.9 (Dr. Maisch; Kovalchuk et al., 2019). The samples were eluted with a linear gradient of 80% ACN, 19.9% H_2_O, 0.1% FA (buffer B) in 99.9% H_2_O and 0.1% FA (buffer A) from 8 to 50% of buffer B in 30 min at 0.5 μL/min flow. For each sample type, three biological replicates and two to three technical sample preparation replicates were analyzed. MS data were collected in DDA (Data Dependent Acquisition) mode for spectra library generation and DIA (Data Independent Acquisition) mode for peptide and protein quantitation. For comprehensive peptide library generation, one sample from each biological replicate was run in the DDA mode. The samples were run using three slightly different DDA methods for better total peptide identification coverage. The MS1 parameters were as follows: 120,000 resolution, 350–1010 scan range, standard AGC target and auto maximum injection time. Ions were isolated with 1.6 m/z window targeting the highest intensity peaks of +2 to +7 charge and a 5 × 10^4^ intensity threshold. Dynamic exclusion was set to 20 s. MS2 fragmentation was carried out in the HCD (Higher energy Collisional Dissociation) mode at 7,500 resolution with 30% NCE (Normalized Collision Energy). The mass range was set to normal, scan range to auto and AGC target to standard. The maximum injection time was set to 18 ms. The total cycle time was set to 2 s. The second DDA method used 10 ms dynamic exclusion and the third method used 20 s dynamic exclusion but 15,000 MS2 resolution and 22 ms maximum injection time. All the samples were run in a single LC-MS DIA run. The DIA parent ion mass ranged 350–1010 m/z divided into 45 windows 14 Da wide. The MS2 resolution was set to 7,500 and the maximum injection time was set to 18 ms. The rest of the parameters were set to default values.

#### Data Processing

Identification of the DDA files was performed with the MaxQuant 1.6.6.0 Software with default settings against the *M. gallisepticum S6* Uniprot reference database. The resulting list of peptides was used to create a spectral library using the Skyline software. Further analysis of the DIA files was performed in the Skyline software by using the default DIA protocol. Retention times were aligned using a built-in iRT calculator and DDA files. The same *M. gallisepticum S6* Uniprot database was used to create a transition list. Quantitative analysis was performed using a default quantification protocol. The resulting quantification data were normalized by equalizing run medians. The proteomic data is available via PRIDE database, project ID PXD019077.

### Gene Cloning, Mutagenesis, and Protein Purification

Recombinant proteins were obtained as previously described (Fisunov et al., 2016a). The genes *pgi, pfkA, fba, tpiA, gapd, pgk, gpmI, eno, pykF*, and *ldh* were amplified from *M. gallisepticum S6* genomic DNA and cloned into the pET15 plasmid with an N-terminal His-tag and thrombin cut site by using *Bam*HI and *Sal*I sites. Tryptophan codon TGA was edited to TGG by Polymerase Chain Reaction (PCR). The primers used are listed in Supplementary Table 1. All genetic engineering experiments were carried out using the Top-10 strain of *E. coli*. The proteins were overexpressed in *E. coli* BL21-Gold (DE3) cells. Proteins were obtained in a water-soluble form. Cells from an overnight culture were harvested using centrifugation, washed in PBS and lysed in a sample buffer (20 mM Na_2_HPO_4_, 10 mM imidazole and 500 mM NaCl pH 7.5) using sonication and a Branson 250 Sonifier (Branson) at 22 kHz for 10 min. The lysate was diluted with the sample buffer. The protein was purified on a Tricorn 5/50 column (GE Healthcare) with Ni Sepharose High Performance (GE Healthcare) resin by using the AKTA FPLC system (GE Healthcare). After the application of the lysate, the column was washed with 25-mL aliquots of the sample buffer. After that the column was washed with wash buffer (20 mM Na_2_HPO_4_, 25 mM imidazole, 500 mM NaCl and pH 7.5) and finally it was washed with elution buffer (20 mM Na_2_HPO_4_, 500 mM imidazole, 500 mM NaCl and pH 7.5) to obtain the recombinant protein. The proteins were stored at −20°C in 50% glycerol.

### Electrophoretic Mobility Shift Assay

FAM-labeled DNA fragments were obtained using PCR from *M. gallisepticum* genomic DNA. The primers used are listed in Supplementary Table 1. P_*eno*, P_*gapd* and P_*rplJ* amplicons corresponded to the promoter regions of the respective operons. Forward primers were used for 5’-6-FAM modification. An aliquot of purified protein was incubated with 2.5 pmol of the FAM-labeled DNA fragments for 10 min at 37°C. Binding reactions with the proteins were performed in 20 mM carbonate buffer (pH 9.8) with 6% glycerol. Electrophoresis was performed using a Mini-PROTEAN Tetra electrophoretic cell (Bio-Rad) and 6% acrylamide gel for 1 h at 10 mA and 10°C. Gels were visualized using a ChemiDoc MP imaging system (Bio-Rad). The fluorescence was quantitated using Image Lab 5.1 software (Bio-Rad). The Kd value for enolase was calculated using the fractional saturation data (protein-shifted band fluorescence to the total fluorescence ratio) and the Hill model Fraction bound = 1/{1+(KD/[Eno])^*n*^}, where n is the Hill coefficient.

### Atomic Force Microscopy

For the preparation of a GM-HOPG surface, 10 μL of 0.01 g/L [Gly_4_-NHCH_2_]_2_C_10_H_20_ (GM, Nanotuning, Russia) solution in water was deposited onto a freshly cleaved HOPG (ZYB quality, mosaic spread 0.8-1.2°, NanoAndMore, Switzerland and NT-MDT, Russia) surface for 10 min, then supplemented with 100 μL of Milli-Q water and dried with a nitrogen flow. The enolase mixture containing DNA was prepared in 20 mM Tris-HCl buffer at pH 7.5. The DNA fragments were obtained using PCR for Electrophoretic Mobility Shift Assay (EMSA). For the Atomic Force Microscopy (AFM) study under ambient conditions, 0.5 μL sample solution was deposited onto a GM-HOPG surface for 1 s, followed by the addition of 100 μL of deionized water for 10 s. Subsequently, the droplets were removed through nitrogen flow. Atomic force microscopy images were acquired under ambient conditions using a multimode atomic force microscope, Ntegra Prima (NT-MDT, Russia), operated with ultrasharp tips (carbon nanowhiskers with a curvature radius of several nanometers grown at tips of commercially available silicon cantilevers with a spring constant of 5-30 N/m) in an attraction regime of intermittent contact mode (Cameron et al., 2011). The line scan rate was typically 1 Hz with 1024 × 1024 pixels per image. The images were analyzed using Nova software (NT-MDT, Russia). The length of DNA loops of the nucleoid was measured using DNA Trace software. For the analysis of enolase AFM images, grains were marked by threshold (all structures with z values below 1.8 nm were discarded) and their geometry was assessed using Gwyddion SMP data analysis tools.

### RNA Purification and cDNA Synthesis

RNA was isolated as previously described (Gorbachev et al., 2013). One-hundred-microliter aliquots of *M. gallisepticum* culture were directly lysed in TRIzol LS reagent (Life Technologies) at a 1:3 ratio of culture medium: TRIzol LS (v/v). The nucleic acids were extracted with chloroform and precipitated by the addition of an equal volume of isopropanol followed by centrifugation (16,000 g, 15 min). The pellets were washed with 80% ethanol and finally resuspended in 20 μL of Milli-Q (Panreac). The amount of RNA was determined using a Qubit 2.0 fluorometer (Thermo Fisher Scientific). The resulting RNA was treated with DNAse I (Thermo Scientific) and cDNA was synthesized from random hexamer primers by using Maxima H Minus Reverse Transcriptase (Thermo Scientific) according to the manufacturer’s protocol.

### Quantitative Real-Time Polymerase Chain Reaction

Quantitative real-time PCR was performed using dNTP, PCR buffer, Taq-polymerase (Lytech), SYBR Green I (Invitrogen) and CFX96TM Real-Time PCR Detection System (Bio-Rad). The primers used are listed in Supplementary Table 1. All primers were designed using BAC-Browser (Garanina et al., 2018). Each 20-μL reaction contained 0.2 μL of template cDNA. The thermal cycling conditions were as follows: initial denaturation at 95°C for 1 min; then 40-cycle amplification (94°C for 15 s, 58°C for 20 s and 68°C for 1 min). The melting curve was obtained by gradually heating the PCR mixture from 65 to 94°C at a rate of 0.5°C every 5 s, with continuous fluorescence scanning. The relative expression for each sample was determined using the 2–ΔΔCt method and normalized to the amount of *eno* transcripts present in the RNA samples. qRT-PCR experiments were performed on two biological replicates per transformant.

### Fluorescence Assay

For the fluorescence assay, 1 mL of a mild-log culture of *M. gallisepticum* (12 h of growth) was harvested by centrifugation at 9000 rpm and 4°C for 10 min. The supernatant was removed and the cells were washed twice with a wash buffer (150 mM NaCl, 50 mM Tris, 2 mM MgCl_2_ and pH 7.4) under sterile conditions. The pellets were resuspended in 100 μL of the same wash buffer and transferred to a 96-well plate. Fluorescence was detected using a CLARIOstar Multi-mode Microplate Reader (BMG LABTECH) at 505 nm after 10 min of incubation at 37°C. The control samples included wild-type *M. gallisepticum* and the wash buffer.

## Results and Discussion

### The Structure and the Protein Content of the *M. gallisepticum* Nucleoid Are Different in the Exponential and Stationary Growth Phases

Our previous transcriptome analysis of *M. gallisepticum* revealed widespread transcriptional suppression upon transition to the stationary phase (Mazin et al., 2014). In theory, this effect may be explained by different mechanisms. First, this may be due to ATP depletion (Buckstein et al., 2008), which can lead to the dissipation of negative supercoiling, which in turn is important for effective transcription initiation. At the same time, a low concentration of NTPs slows down transcription elongation. In addition, protein-mediated compaction and blocking of genomic DNA may also occur (Gray et al., 2020; Scholz et al., 2020). This process may functionally resemble the process of heterochromatinization in eukaryotes. To study this, we performed both structural and proteomic analyses of the *M. gallisepticum* nucleoids in the exponential and stationary phases.

**FIGURE 1:**
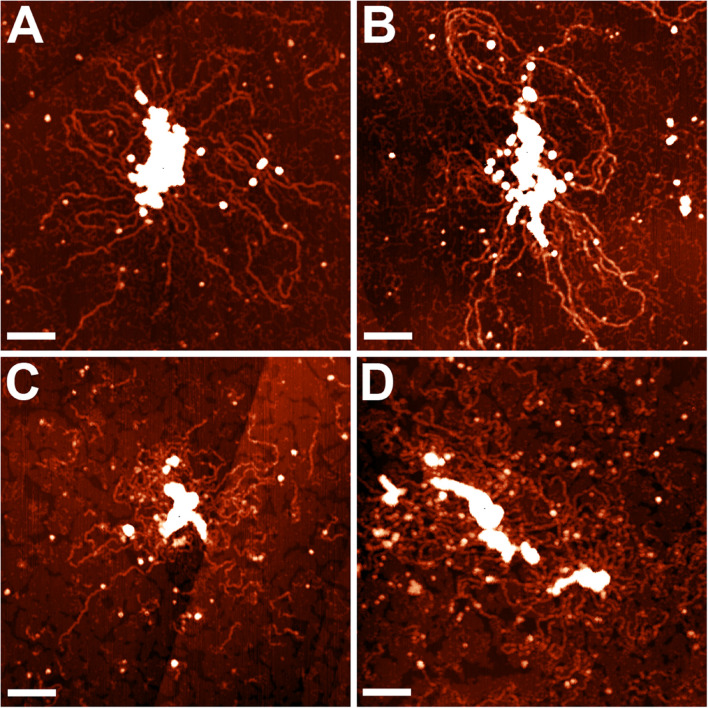
AFM images of the *M. gallisepticum* nucleoid structural units at the exponential **(A,B)** and the stationary **(C,D)** growth phases. Bar = 100 nm.

**FIGURE 2:**
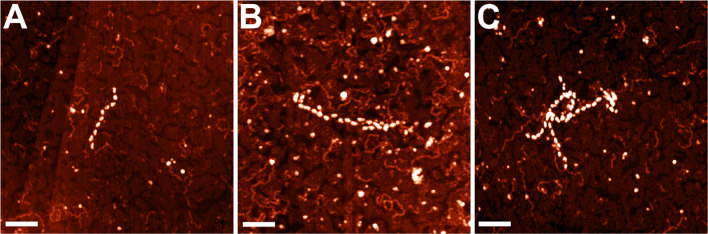
AFM images of the structures **(A–C)**, observed only in the stationary phase nucleoids of *M. gallisepticum*. Bar = 100 nm.

The nucleoid fractions of *M. gallisepticum* were isolated from synchronized cell cultures by centrifugation in a sucrose gradient. After isolation, the fractions were visualized using AFM The nucleoid fractions isolated from the exponential and stationary cells showed differences at the structural level (Figure 1 and Supplementary Figures 1, 2). Nucleoids from the exponential phase cells formed structures with dense protein-enriched cores with DNA loops protruding from the core. The length of the loops was approximately 200–600 nm (Supplementary Figure 3), which, taking into account the base pair length as 0.34 nm, resulted in 1–2 kilobases per loop, which is comparable to the average gene size in *M. gallisepticum*. The stationary phase nucleoids showed compaction compared to the exponential phase nucleoids (Figure 1). The free DNA loops were shorter (approximately 100–200 nm, Supplementary Figure 3) and more populated with proteins. The observed DNA loops were shorter than CIDs (Chromosomal Interaction Domains) calculated form Hi-C data for another minimal bacterium, *Mycoplasma pneumoniae* (Trussart et al., 2017). CIDs identified for *M. pneumoniae* ranged from 15 to 33 kb or from 5 to 11 μm. The stationary phase nucleoids featured unique structures that resembled bead chains formed by proteins regularly bound along DNA strands (Figure 2). The average bead length was 16-20 nm (Supplementary Figure 4). The lengths of these structures were in the range of 200–400 nm. Thus, they represent several hundred bases to kilobase fragments of DNA and do not represent promoters or other short regulatory sequences. In general, the stationary phase nucleoids appeared denser than the exponential phase nucleoids.

**FIGURE 3:**
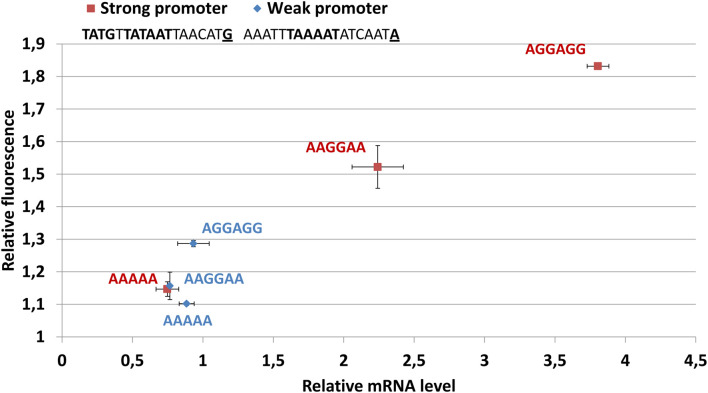
The abundance of mMaple2 mRNA and protein produced in genetic constructs with different strengths of promoter and ribosome-binding site. The key promoter determinants (EXT-element, -10-box, and initiator nucleotide) are underlined and highlighted in bold. The ribosome binding sites are shown on the chart in red for strong promoter constructs and in blue for weak promoter constructs.

To reveal the physical basis of this rearrangement, we performed LC-MS/MS proteomic analysis of the isolated nucleoid fractions (Supplementary Table 2 for the exponential phase and Supplementary Table 3 for the stationary phase). We used the total *M. gallisepticum* proteome as a control. The nucleoid and cytoplasmic compartments of the bacteria are not physically isolated. Thus, there is an affinity-guided dynamic equilibrium between these compartments. The amount of a particular protein within the nucleoid fraction depends on both the binding constants and concentrations. The major cellular proteins can associate with the nucleoid (DNA or NAPs) non-specifically and thus provide a high noise signal, masking the signal of more specific but less abundant proteins. At the same time, the enrichment data provide limited information. For example, conditionally controlled transcription factors may be highly enriched under certain conditions, but are very low in abundance and thus, not responsible for global structural rearrangements. Thus, we analyzed nucleoid-associated proteomes in two dimensions: protein enrichment relative to the unfractionated lysate and relative abundance of the proteins within the nucleoid-associated fraction (Figure 3). We proposed that the proteins involved in the structural maintenance of the chromosome have to be highly abundant and significantly enriched in the nucleoid-associated fraction. At the same time regulators, such as transcription factors, are low-copy proteins. Therefore, we expected them to be enriched but low in abundance.

**FIGURE 4:**
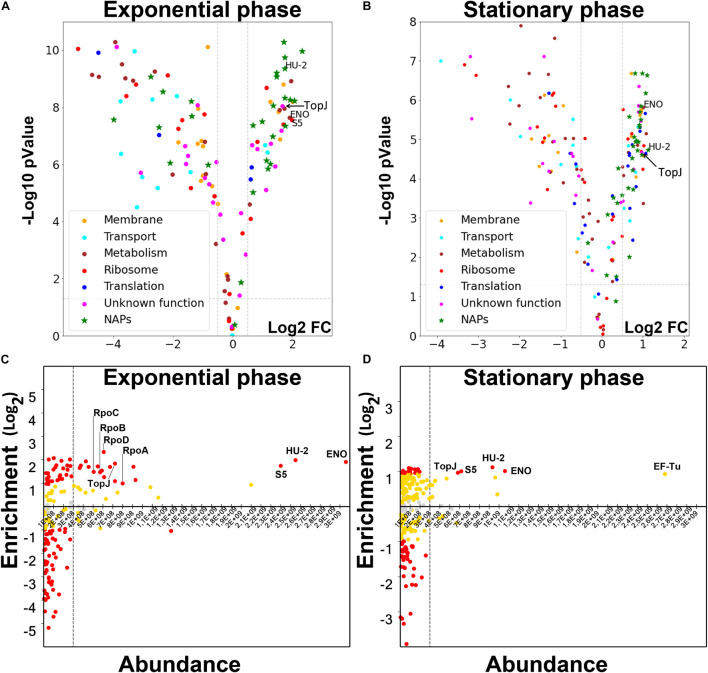
**(A,B)** Volcano plots of the protein enrichment data for *M. gallisepticum* nucleoid in the exponential **(A)** and the stationary **(B)** growth phases. Putative NAPs were predicted based on homology with known proteins. Cutoffs (log_2_ fold change ± 0.5 and p-value = 0.05) are shown as dashed lines. **(C,D)** The distribution of the identified NAPs of the *M. gallisepticum* according to their enrichment in the nucleoid fraction compared to lysate and the abundance within the nucleoid fraction in the exponential **(C)** and the stationary **(D)** growth phases. Vertical axis represents Log_2_ fold change of the protein abundance in the nucleoid fraction compared to the total lysate (the positive values indicate enrichment in the nucleoid fraction, the negative ones indicate depletion). Red dots represent proteins with the statistically significant (p-val < 0.05) enrichment or depletion more than two-fold. Yellow dots represent protein with enrichment or depletion less than two-fold, but still statistically significant (p-val < 0.05). Gray dots represent proteins, which enrichment or depletion is not statistically significant.

The most abundant components of the nucleoid-associated fraction were the HU protein homolog, encoded by the *hup_2* gene (further HU-2), enolase (GCW_02860) and S5 ribosomal protein. This was observed in both the exponential and stationary phase nucleoids (Figure 4). While the HU protein is a conserved constituent of a bacterial chromosome, the role of enolase as a NAP was surprising. Generally, the dynamic range of protein enrichment was higher and the repertoire of the enriched proteins was greater in the exponential phase nucleoids. The proteins enriched in the exponential phase nucleoids can be classified into three distinct groups: highly abundant (HU-2, enolase, S5), medium-abundant and low-abundant. We classified as low-abundant proteins, those proteins whose abundance was not higher than that of the non-enriched proteins (e.g., non-NAPs). The medium-abundant group included 16 proteins: all four subunits of RNA polymerase, S3 and S4 ribosomal proteins, components of adhesive apparatus, or the terminal organelle (TopJ, GapA, CrmA, PlpA, HMW-1, and Hlp-3), PepC-1 protease, ClpB chaperone, SMC homolog GCW_01885 and the HAD-hydrolase family protein GCW_01555.

Mycoplasmas feature a specialized structure for motility as well as host cell adhesion and penetration, called terminal, attachment, or tip organelle (Hasselbring et al., 2006; Miyata, 2008). Among the proteins of the terminal organelle, GapA and CrmA featured predicted export signals and a transmembrane region. Both proteins also contained cytoplasmic domains. The remainders were purely cytoplasmic components of the terminal organelle. The data obtained may indicate that the cytoplasmic components of the tip organelle may tightly interact with DNA at certain loci. Terminal organelle duplication is tightly linked with cytokinesis (Hasselbring et al., 2006). Thus, we hypothesize that genomic DNA is bound to the intracellular part of the terminal organelle at the origin of replication. This hypothesis corroborates previous observations that the genomic DNA of *M. gallisepticum* associates with the membrane and, in particular, the terminal organelle (Quinlan and Maniloff, 1972).

*Mycoplasma gallisepticum* features two homologs of the SMC protein. One is a full-length homolog (GCW_90999) and the other is a shortened variant (GCW_01885) that lacks a significant part of the C-terminus. The shortened variant was found within the medium-abundant group, whereas the full-size homolog was not detected. One can speculate that the full-size SMC homolog may be recruited to DNA only during cell division, while the GCW_01885 functions constitutively as a chromosome structural protein. The truncation of DNA-binding domain of GCW_01885 may be compensated by the interaction with a DNA-binding partner.

The proteome content of the stationary-phase nucleoids was drastically different from that of the exponential phase nucleoids (Figure 4). First, the repertoire of NAPs was significantly scarcer. HU-2, enolase and S5 ribosomal proteins were retained in the major NAP group. Additionally, TopJ protein, which was abundant in the exponential phase, became a major protein. The group of medium-abundant proteins completely disappeared and the respective proteins became less abundant. This includes all the subunits of RNA polymerase. They were both less enriched and less represented. The observed effect raises the question of whether it is a cause or a consequence of genome-scale transcriptional repression at the stationary phase. At least, the proteomic content of the nucleoid, transcriptomics data and structural data demonstrated good correlation. We propose that genome-scale reorganization of transcription can be mediated only by major structural proteins or global processes, such as the degree of supercoiling. The repertoire of the major NAPs did not show significant changes, except TopJ, which became a major NAP. Thus, we propose that global transcriptional repression and nucleoid compaction may occur via rearrangement of existing NAPs. It may also be modulated by the TopJ. Stationary phase nucleoids feature one more major protein, EF-Tu. This protein features the highest abundance, but a moderate enrichment, which is below our cutoff (2-fold enrichment, p-value < 0.05). However, EF-Tu demonstrated remarkable dynamics between the two growth phases. In the exponential phase it was significantly depleted, probably, effectively excluded from the nucleoid. In the stationary phase nucleoid, it became enriched and more abundant, even in comparison with HU-2, enolase and S5. Thus, EF-Tu may be another player in nucleoid structural rearrangement in the stationary phase.

The observed distribution of ribosomal proteins leads to the conclusion that some of them serve as NAPs (Figure 3). If we isolated ribosomes associated with nucleoids, one could expect a more even distribution of ribosomal proteins. However, a few of them were highly abundant as NAPs, while others were significantly depleted. One can speculate that ribosomal proteins bind DNA non-specifically because of the highly alkaline pI. However, some ribosomal proteins can bind simple RNA structures and may serve as riboswitch ligands (Deiorio-haggar et al., 2013). Thus, some of the enriched ribosomal proteins, including S3, S4, and S5, may bind not only the chromosome itself, but also the nucleoid-associated transcripts.

Transcription factors represent a distinct group of NAPs. On one hand, they feature high sequence specificity and, as a result, a limited number of binding sites per genome. On the other hand, their copy number per cell is generally lower than that of other NAPs. This makes the identification of TFs on the background of major NAPs a difficult task. However, we were able to identify some TFs or TF-like proteins (homologous to TFs but with unclear binding sites) in our proteomic data. We detected HrcA (heat-shock repressor), GntR-like protein (GCW_02645) and YebC/PmpR homolog (GCW_00725). HrcA was significantly enriched in the exponential phase nucleoids, but not in the stationary phase nucleoids, which corroborates the activation of its target genes encoding chaperones ClpB and DnaK, according to the transcriptomic data. The GntR homolog was enriched only in the exponential phase nucleoids, while the YebC/PmpR homolog was enriched only in the stationary phase nucleoids. The transcriptomic data indicated massive transcriptional repression in the stationary phase. However, a limited set of genes undergo significant activation. Thus, one can speculate that YebC/PmpR may serve as an activator of some of these genes.

Based on the data obtained, we studied the distribution of molecular machines responsible for transcription, translation and mRNA degradation in exponential phase nucleoids. All RNAP subunits were found in the medium-abundance group of proteins. Ribosomal proteins demonstrated very different enrichment patterns. Most of them were not enriched in the nucleoid fraction. The general conclusion is that ribosomes form a different compartment and the well-studied phenomenon of transcription-translation coupling is not a major pathway of gene expression in *M. gallisepticum*. Two RNase J homologs, GCW_00095 and GCW_00450, were found within the low-abundance group of proteins. These enzymes are multifunctional, but in the gram-positive clade, they play a key role in general mRNA turnover (Quinlan and Maniloff, 1972). Thus, we propose that the mRNA degradation function is associated with the nucleoid. However, some nucleases may also be present in the cytoplasm. Previously, we found that the heat stress of *M. gallisepticum* drastically perturbs its transcriptional landscape, but the ribosome-bound fraction of mRNA undergoes very limited changes (Fisunov et al., 2017). This observation corroborates the hypothesis that ribosomes form a different compartment that may, to some extent, maintain homeostasis independently of the chromosome and transcription machinery. To test this hypothesis, we assembled a set of reporter constructs in which the mMaple2 coding sequence (Rumyantseva et al., 2019) was transcribed from a strong or weak promoter and featured different types of Shine-Dalgarno (SD) sequences: strong consensus (AGGAGG), weak (AAGGAA) and none (AAAAA). The translational determinants of the 5’-ends of *M. gallisepticum* mRNAs have been previously reported in Mazin et al. (2014) and Fisunov et al. (2017). The actual promoter-SD DNA blocks are listed in Supplementary Table 1. The codon usage frequencies of the mMaple2 coding sequence were optimized for *M. gallisepticum* to ensure effective translation. For the construct with a strong promoter, we observed a direct correlation between mMaple2 mRNA abundance and the strength of the SD sequence (Figure 3). A direct correlation between mRNA abundance and mMaple2 fluorescence was observed as well. The construct with a weak promoter showed no correlation between mRNA abundance and SD sequence strength. For this series of constructs, the mRNA levels were always low (as well as the fluorescence). We hypothesize that the increase in mRNA abundance solely by the enhancement of the SD sequence may occur through the more effective escape from nucleoid compartment. The mRNA with strong translational determinants effectively leaves the nucleoid compartment, where RNase activity is localized and enters the ribosome compartment, where nuclease activity is depleted. We propose that the limiting step for the weak promoter is mRNA production. It significantly degrades prior to entry into the ribosome compartment.

### Enolase Is a Novel Nucleoid-Associated Protein in *Mycoplasma gallisepticum*

**FIGURE 5:**
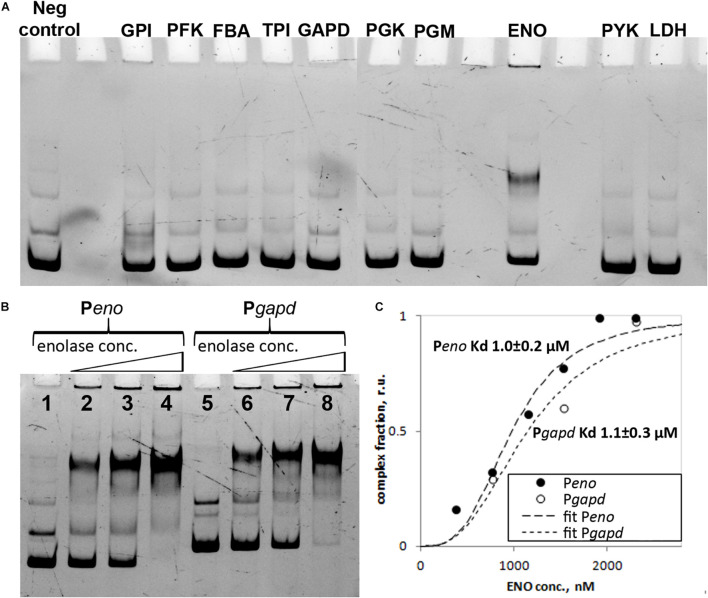
**(A)** Electrophoretic mobility shift assay (EMSA) of the DNA-binding activity of glycolysis proteins from *M. gallisepticum*, enolase gene promoter region was used as a probe (P*eno*), 250 nM: glucose-6-phosphate isomerase (GPI) 879 nM, 6-phosphofructokinase (PFK) 863 nM, fructose-bisphosphate aldolase (FBA) 2255 nM, triosephosphate isomerase (TPI) 1605 nM, glyceraldehyde-3-phosphate dehydrogenase (GAPD) 127 nM, phosphoglycerate kinase (PGK) 1589 nM, phosphoglycerate mutase (PGM) 1038 nM, enolase (ENO) 769 nM, pyruvate kinase (PYK) 332 nM and L-lactate dehydrogenase (LDH) 1155 nM. **(B)** EMSA of *M. gallisepticum* enolase with different DNA fragments including promoter regions of enolase (P*eno*) and glyceraldehyde-3-phosphate dehydrogenase (P*gapd*) genes. Lanes: 1 – free DNA fragment (P*eno*), 2 – P*eno* + 769 nM of enolase, 3 – P*eno* + 1539 nM of enolase, 4 – P*eno* + 2308 nM of enolase, 5 – free DNA fragment (P*gapd*), 6 – P*gapd* + 769 nM of enolase, 7 – P*gapd* + 1539 nM of enolase, 8 – P*gapd* + 2308 nM of enolase. **(C)** Identification of *M. gallisepticum* enolase (ENO) binding constant using protein titration in EMSA experiment and Hill equation. P*eno* DNA fragment was used for the binding constant determination.

The data on protein enrichment in the nucleoid-associated fraction demonstrated that enolase is highly abundant and overrepresented in the nucleoids of both the exponential and stationary phases. Glycolysis represents the only way of energy metabolism in *M. gallisepticum* and the respective enzymes are highly expressed and are the major cellular proteins. Among the glycolytic enzymes, only enolase showed substantial enrichment in the nucleoid-associated fraction. Enolase features the most basic pI among the rest of the glycolytic proteins (Supplementary Table 4). Thus, the observed enrichment could be due to non-specific electrostatic interactions. To test the DNA-binding abilities of the glycolytic proteins we cloned all of them and tested their DNA-binding activity using EMSA. We used 10 glycolytic enzymes, starting from glucose-6-phosphate isomerase to lactate dehydrogenase (Figure 5A). Only enolase showed substantial DNA-binding activity. The DNA-binding activity of enolase was tested against different promoter regions of 150–200 bp length including its own enolase gene promoter and glyceraldehyde-3-phosphate dehydrogenase (GCW_01780) gene promoter, lactate dehydrogenase gene promoter, 5′-fragment of 5′-UTR of *rpsD* gene and *tuf* gene promoter (Figure 5B and Supplementary Figure 5). The promoters were precisely mapped in a previous study (Mazin et al., 2014). No differences were identified and thus we concluded that enolase can function as a non-specific DNA-binding protein. The possible sequence or structural preferences remain obscure. Furthermore, we identified the binding constant by using the EMSA titration experiment (Fisunov et al., 2016a) and the Hill equation (Figure 5C and Supplementary Figure 6). For the P*eno* DNA fragment the binding constant was 1.0 ± 0.2 μM and Hill coefficient was 3.1 ± 0.6. For the P*gapd* DNA fragment the binding constant was 1.1 ± 0.3 μM and Hill coefficient was 2.7 ± 0.8.

**FIGURE 6:**
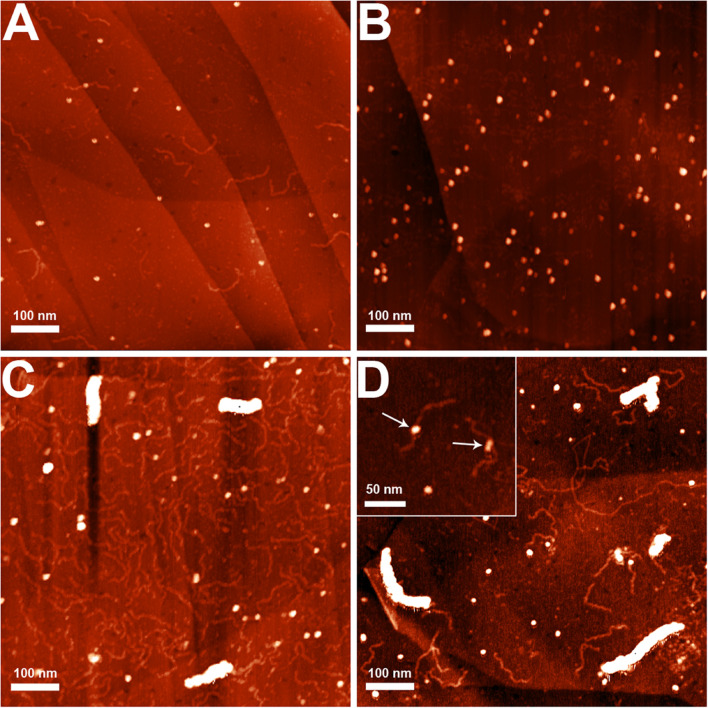
**(A)** AFM images of P*rplJ* DNA fragment, corresponding to *rplJ* operon promoter, 300 bp, **(B)** recombinant enolase preparation, **(C,D)** high-molecular nucleoprotein complexes of enolase with DNA P*rplJ* fragment. Binding reaction was performed same as for EMSA. Panel **(D)** represents 9-fold dilution of DNA in comparison to panel **(C)**. The arrows indicate low-molecular complexes of DNA fragments with enolase.

Furthermore, we used AFM to visualize the complexes of enolase with DNA (300 bp fragments, Figures 6A,B). To distinguish enolase monomers and low-molecular complexes from salt condensates, which are present in all samples, including free DNA samples, we marked them as grains and analyzed their size distribution (Supplementary Figure 7). Median height (Z_med) and zero base volume (V_0) had markedly different distributions in DNA (Figure 6A) and enolase (Figure 6B) samples, confirming that these are distinct species (average Z-med_salt ≈ 2.5 nm and average V_0 salt ≈ 0.55 nm; average Z-med_protein ≈ 3.0 ^∗^10^^^–25m^^^3 and average V_0 salt ≈ 3.1 ^∗^10^^^–25m^^^3). Further analysis using alternative size characteristics (minimum basis volume or Laplasian basis volume) gave similar results.

The observed images showed two types of structures (Figures 6C,D and Supplementary Figure 8). The first represented a low-molecular complex, visualized as small globular structures distributed randomly on DNA (Figure 6D). Probably these complexes are the same as that observed in the EMSA experiments (Figures 5A,B). The second structure comprised high-molecular complexes that formed fibrils approximately 15 nm in width. The length of the fibrils was dependent on the enolase-to-DNA ratio. The increase in DNA concentration resulted in shorter fibrils and vice versa. The formation of a high-molecular complex was observed only in the presence of DNA. Thus, we concluded that the complex assembled on DNA.

The majority of genes in *M. gallisepticum* in the stationary phase undergo repression; few of them do not decrease or even increase transcription (Mazin et al., 2014). Thirty genes were upregulated by more than two-fold in the stationary phase compared to the exponential phase. Among them, only *hup_2* (HU protein) and *dps* (iron storage and DNA protective protein) encode known DNA-binding proteins. The enolase gene was activated by approximately 6-fold, *hup_2* was activated 8-fold and *dps* was activated 16-fold. At the same time, the remaining genes of glycolytic enzymes, except lactate dehydrogenase, were significantly downregulated. Thus, we propose that enolase expression does not increase for the acceleration of the glycolytic pathway. We hypothesize that in *M. gallisepticum*, enolase may play a role in nucleoid protection, integrity maintenance and the global regulation of transcription. However, to carry out the regulatory function, the structure of the enolase complex with DNA has to be modulated by some partners or metabolites, which remain unknown.

It was demonstrated that nucleoids of *Escherichia coli*, *Pseudomonas aeruginosa*, *Bacillus subtilis* and *Staphylococcus aureus* include HU protein in both exponential and stationary growth phases and species-specific NAPs (Ohniwa et al., 2011). *Mycoplasma gallisepticum* lacks substantial amount of NAPs due to the genome reduction. We hypothesize that in mycoplasmas enolase evolved to substitute lacking NAPs. The DNA-binding activity of the enolase enzyme was identified for one of its human homologs α-enolase (Subramanian and Miller, 2000). In this case, a shorter form of α-enolase preferentially accumulates in the nucleus and functions as a specific repressor of the *c-myc* promoter. Thus, DNA-binding may be a more widespread, alternative function of enolase, common in bacteria and eukaryotes.

## Data Availability Statement

The datasets presented in this study can be found in online repositories. The names of the repository/repositories and accession number(s) can be found in the article/Supplementary Material.

## Author Contributions

GF, OP, and VG contributed to design of the study and drafting the manuscript. AZ, OP, MG, TS, SK, and RZ performed nucleoid isolation and proteome analysis. DE performed genetic engineering of *Mycoplasma gallisepticum* and gene expression analysis. VM obtained recombinant enolase from *M. gallisepticum*. GF, OP, and AV performed recombinant enolase activity assay. NB and DK performed atomic force microscopy. All authors contributed to manuscript revision, read, and approved the submitted version.

## Conflict of Interest

The authors declare that the research was conducted in the absence of any commercial or financial relationships that could be construed as a potential conflict of interest.

## Publisher’s Note

All claims expressed in this article are solely those of the authors and do not necessarily represent those of their affiliated organizations, or those of the publisher, the editors and the reviewers. Any product that may be evaluated in this article, or claim that may be made by its manufacturer, is not guaranteed or endorsed by the publisher.
