# Supplementary material for: The Dynamics of Mycoplasma gallisepticum Nucleoid Structure at the Exponential and Stationary Growth Phases
Source: Front Microbiol. 2021 Nov 18;12:753760. doi: 10.3389/fmicb.2021.753760 (PMC8637272; doi:10.3389/fmicb.2021.753760)
Supplement: Supplementary file 1 [file Data_Sheet_1.PDF]

Supplementary figures

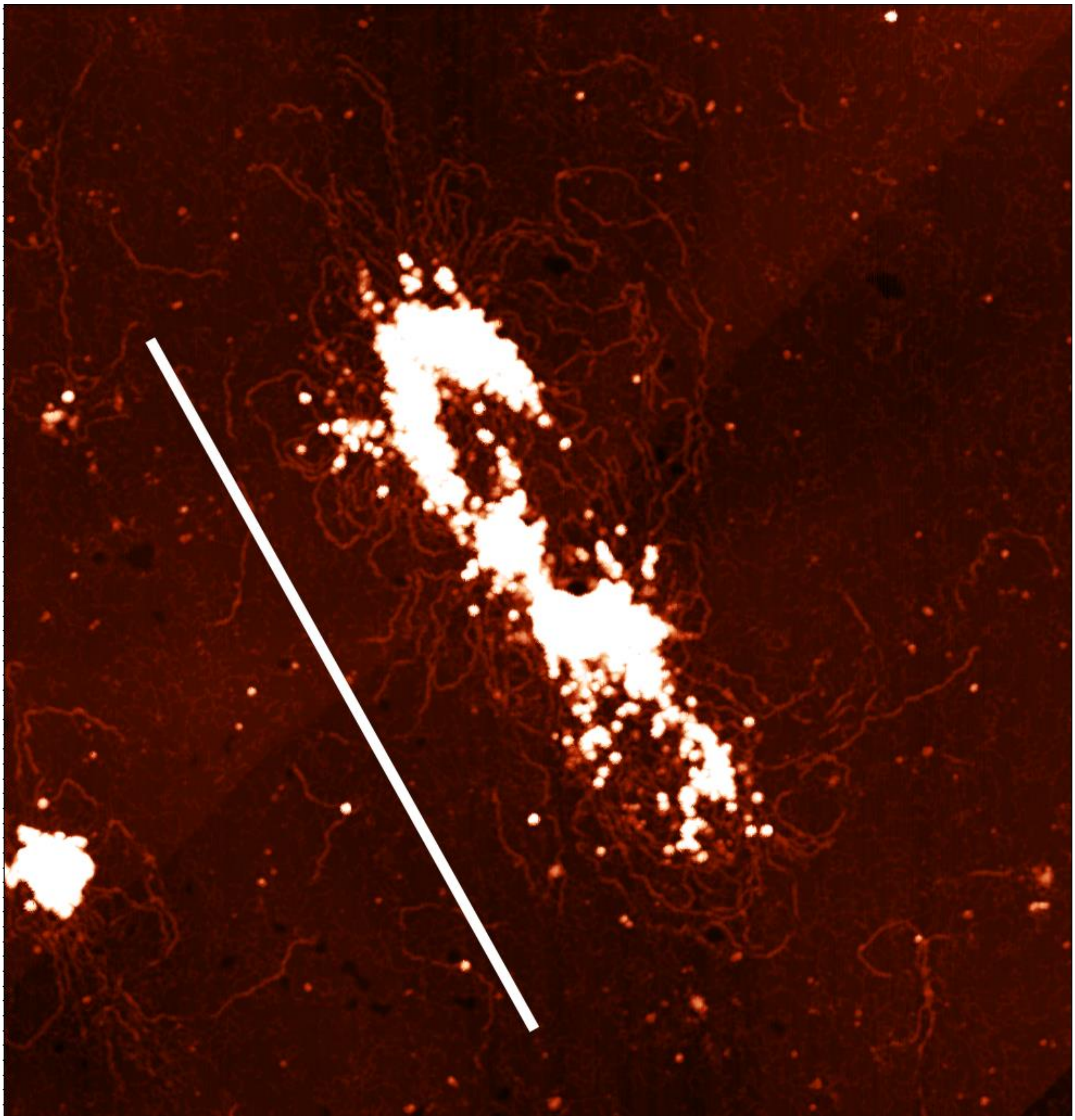

Supplementary figure 1. AFM image of *M. gallisepticum* nucleoid at the exponential growth phase. Bar = 1 µm

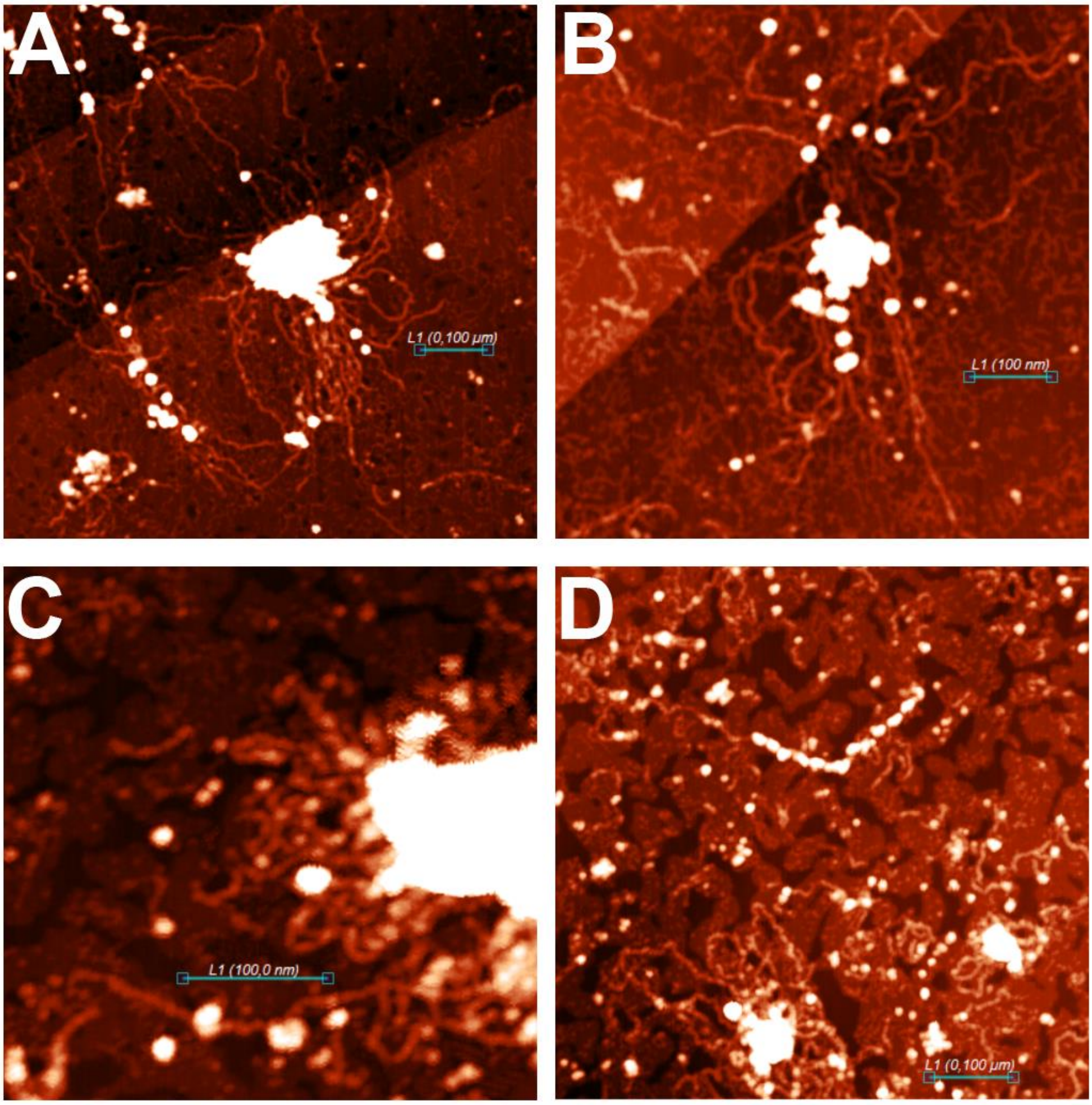

Supplementary figure 2. AFM image of *M. gallisepticum* nucleoid fragments at the exponential (A, B) and stationary (C, D) growth phases. Bar = 100 nm

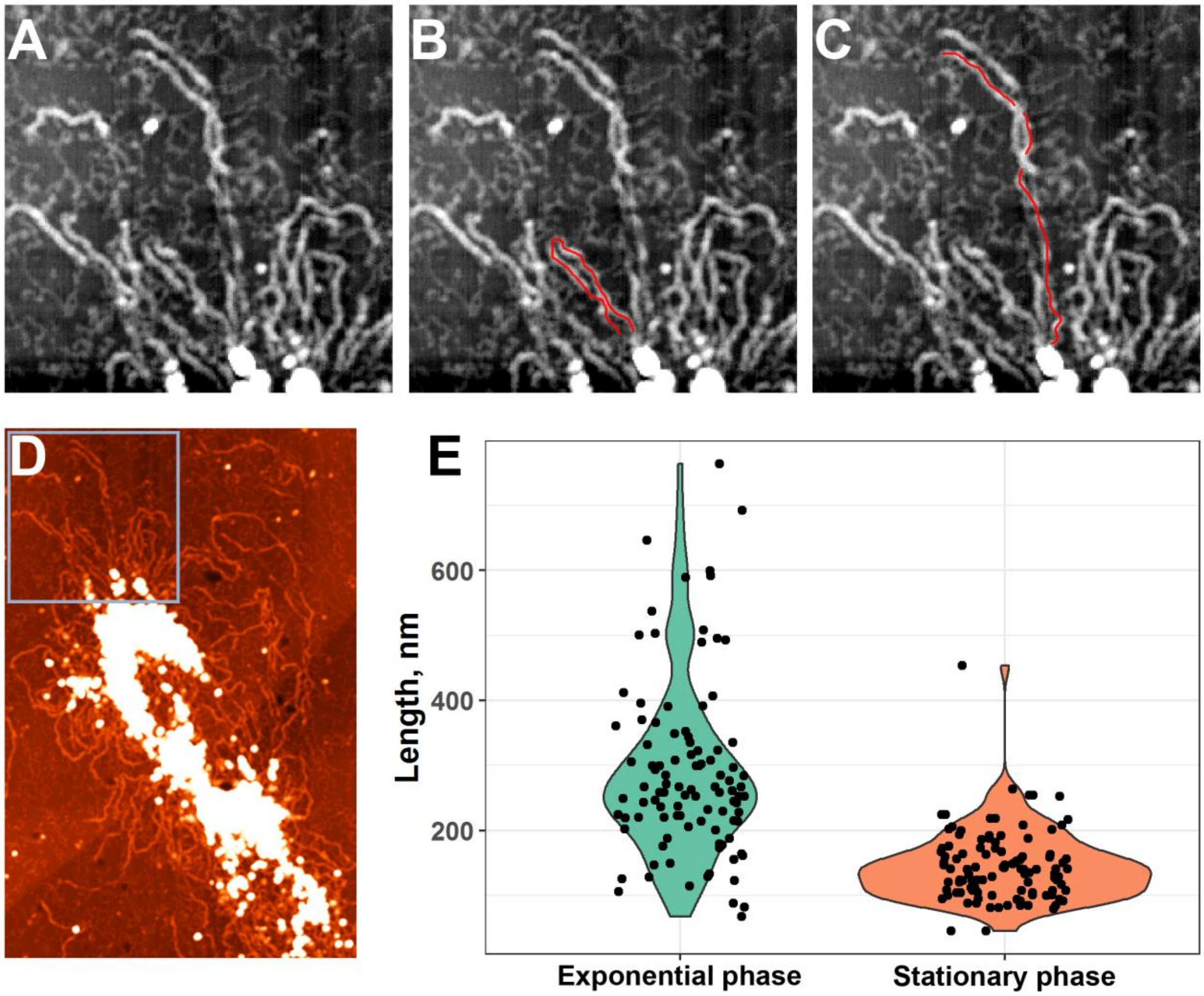

Supplementary figure 3. A – The magnified fragment of nucleoid at the exponential growth phase. The overview of the nucleoid is shown on figure **D**. **B** – Intact DNA loop highlighted in red. **C** – DNA loop possibly broken at its distant end, one of the DNA strands highlighted in red. For this loop there is no clear evidence, that it is indeed one DNA loop broken at its distant end, rather than there are two DNA loops both broken at their proximal ends. Such DNA strands were measured as two separate loops, rather than as one. **E** – The distribution of DNA loops length at the exponential and the stationary growth phases. Exponential phase nucleoids feature on average twice as long DNA loops, p-value<0.01 (Wilcoxon rank sum test with continuity correction).

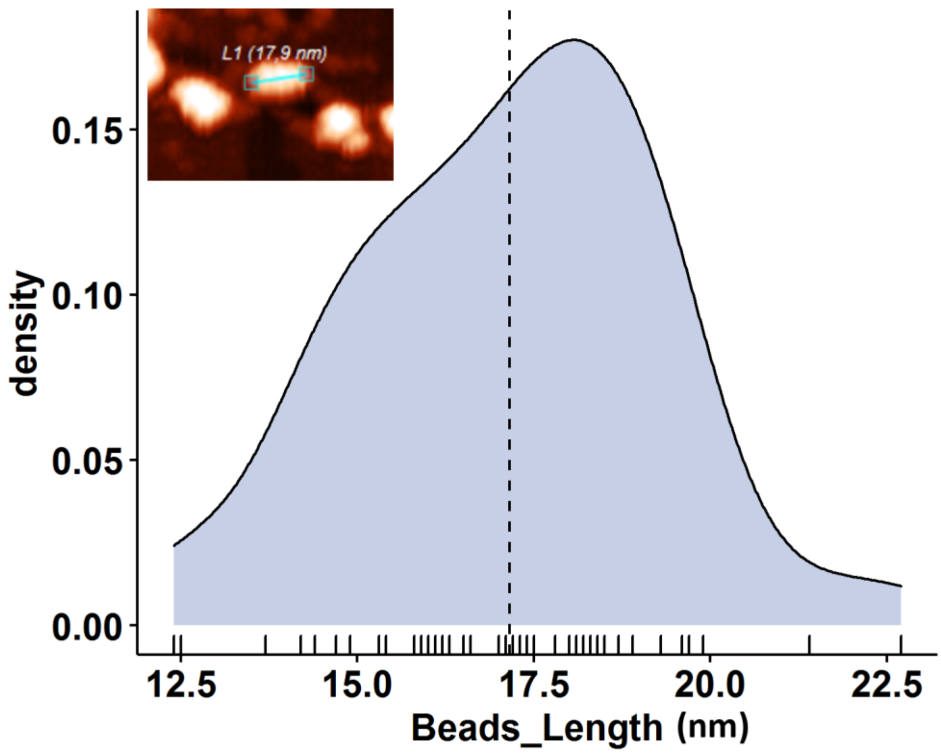

Supplementary figure 4. The distribution of the length of protein beads observed at stationary growth phase and represented on Figure 2.

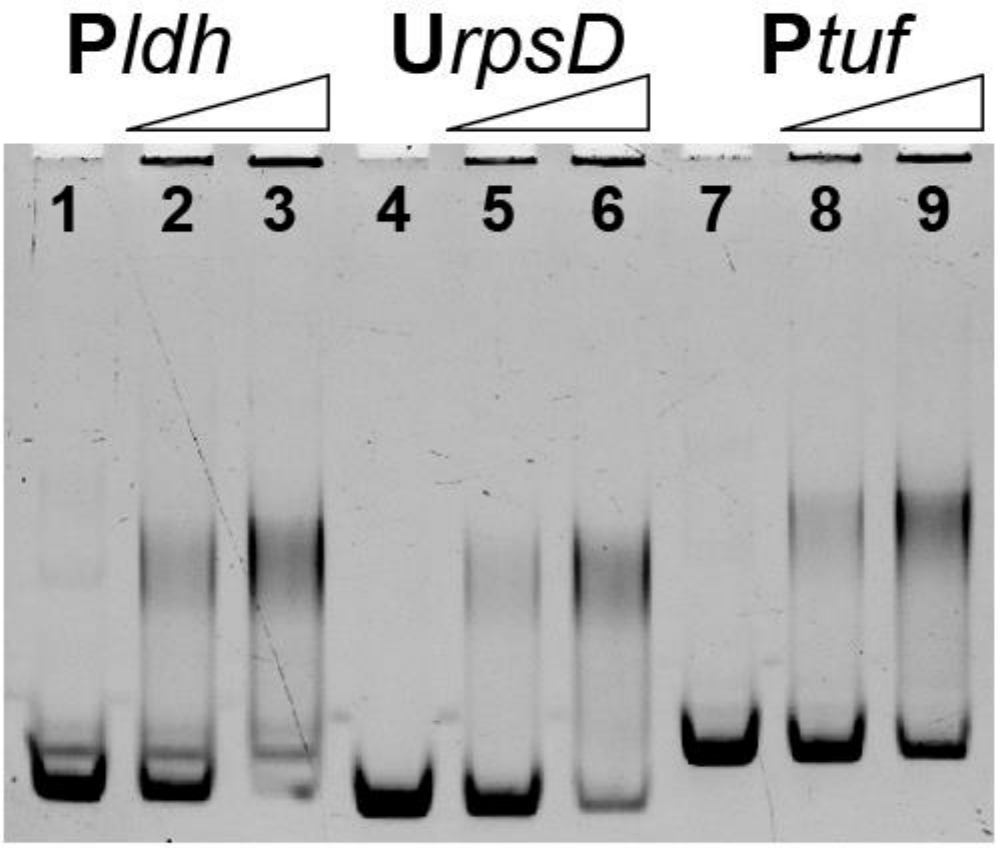

Supplementary figure 5. EMSA of *M. gallisepticum* recombinant enolase with different DNA fragments: promoter region of *ldh* gene (P*ldh*), 5'-fragment of 5'-UTR of *rpsD* gene (U*rpsD*), promoter region of *tuf* gene (P*tuf*). Lanes: 1 – free DNA fragment (P*ldh*), 2 – P*ldh* + 769 nM of enolase, 3 – P*ldh* + 1539 nM of enolase, 4 – free DNA fragment (U*rpsD*), 5 – U*rpsD* + 769 nM of enolase, 6 – U*rpsD* + 1539 nM of enolase, 7 – free DNA fragment (P*tuf*), 8 – P*tuf* + 769 nM of enolase, 9 – P*tuf* + 1539 nM of enolase.

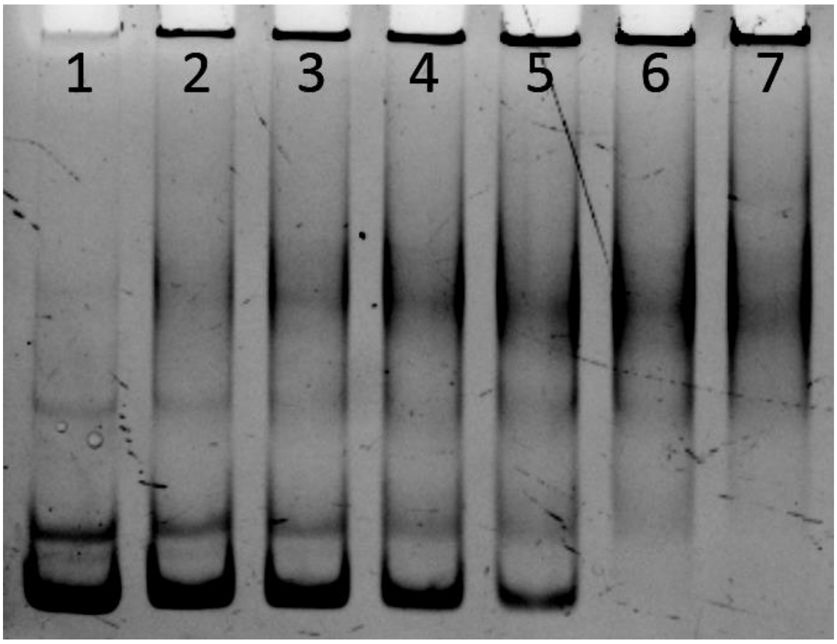

Supplementary figure 6. EMSA of *M. gallisepticum* enolase with P*eno* DNA fragment. Lanes: 1 – P*eno* DNA fragment, 2 – 385 nM of enolase, 3 – 769 nM of enolase, 4 – 1154 nM of enolase, 5 – 1539 nM of enolase, 6 – 1923 nM of enolase, 7 – 2308 nM enolase.

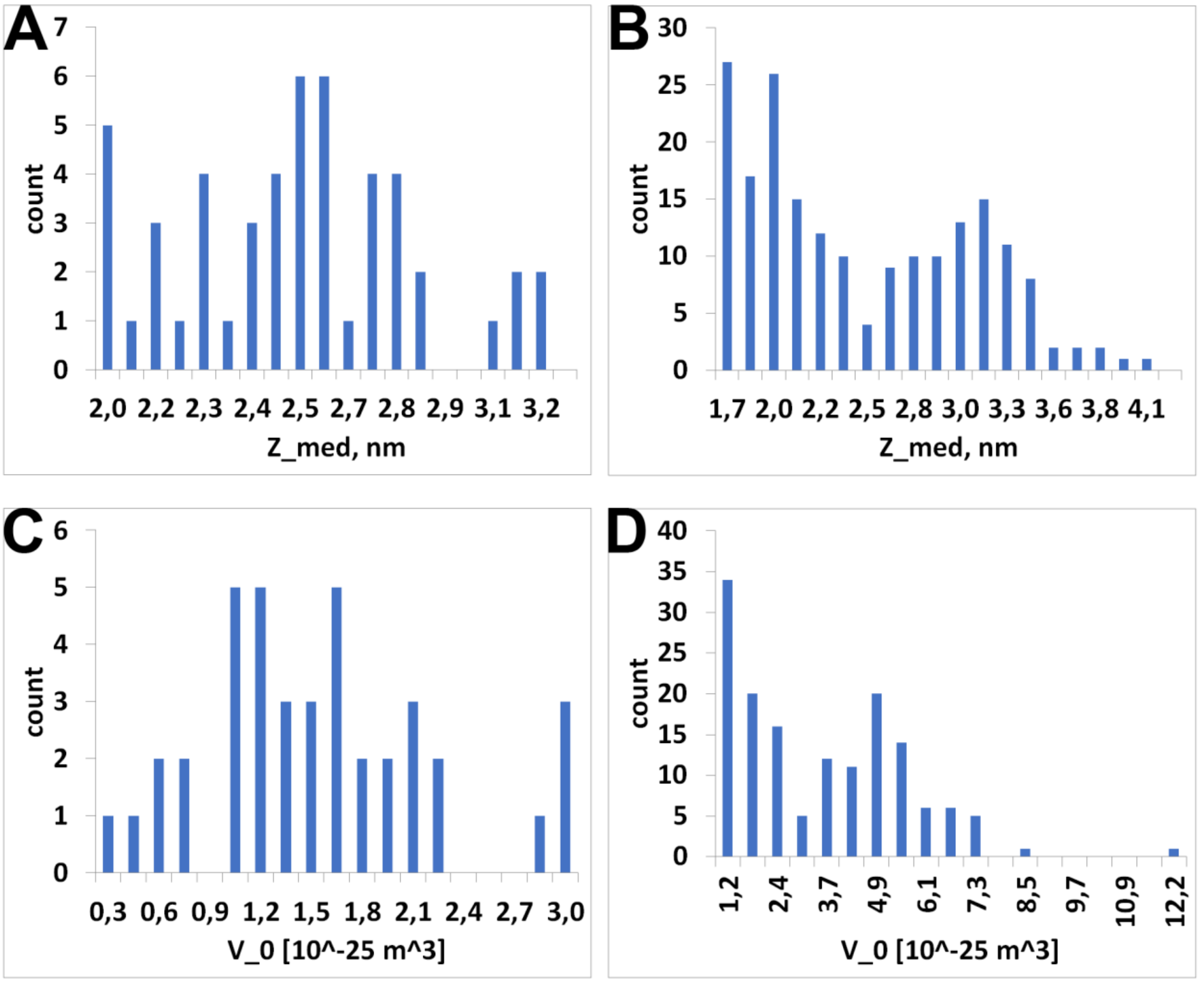

Supplementary figure 7. Analysis of the heights and volumes of particles, represented on Figure 6A, B. The analysis was carried out using Gwyddion SMP data analysis tools. **A** – Height distribution of particles in purified DNA sample, shown on Figure 6A. **B** – Height distribution of particles in recombinant enolase sample, shown on Figure 6B. **C** – Zero base volume distribution of particles in purified DNA sample, shown on Figure 6A. **D** – Zero base volume distribution of particles in recombinant enolase sample, shown on Figure 6B.

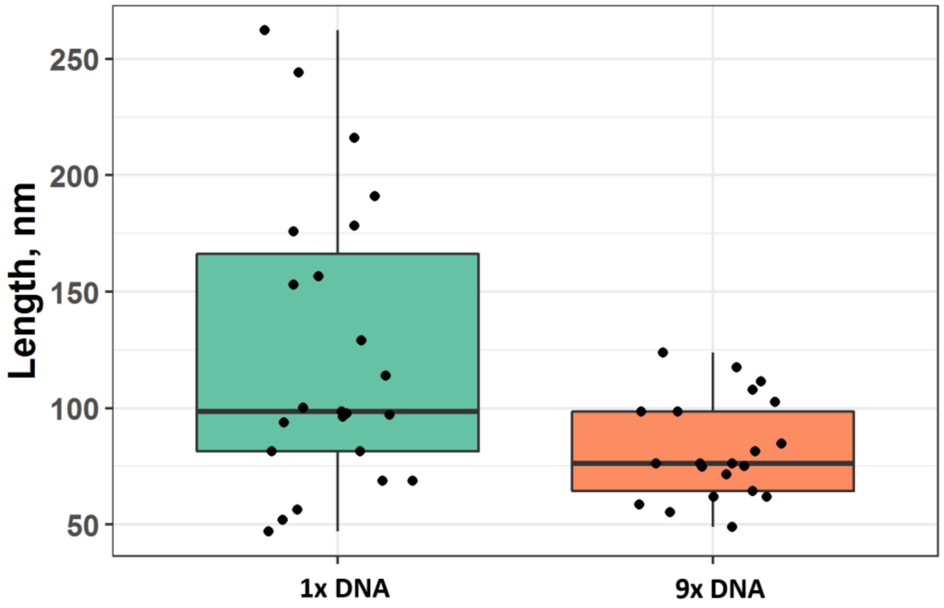

Supplementary figure 8. Length distribution of high-molecular nucleoprotein complexes on Fig. 6C, D. 1xDNA corresponds to Fig. 6D, 9xDNA corresponds to Fig. 6C.

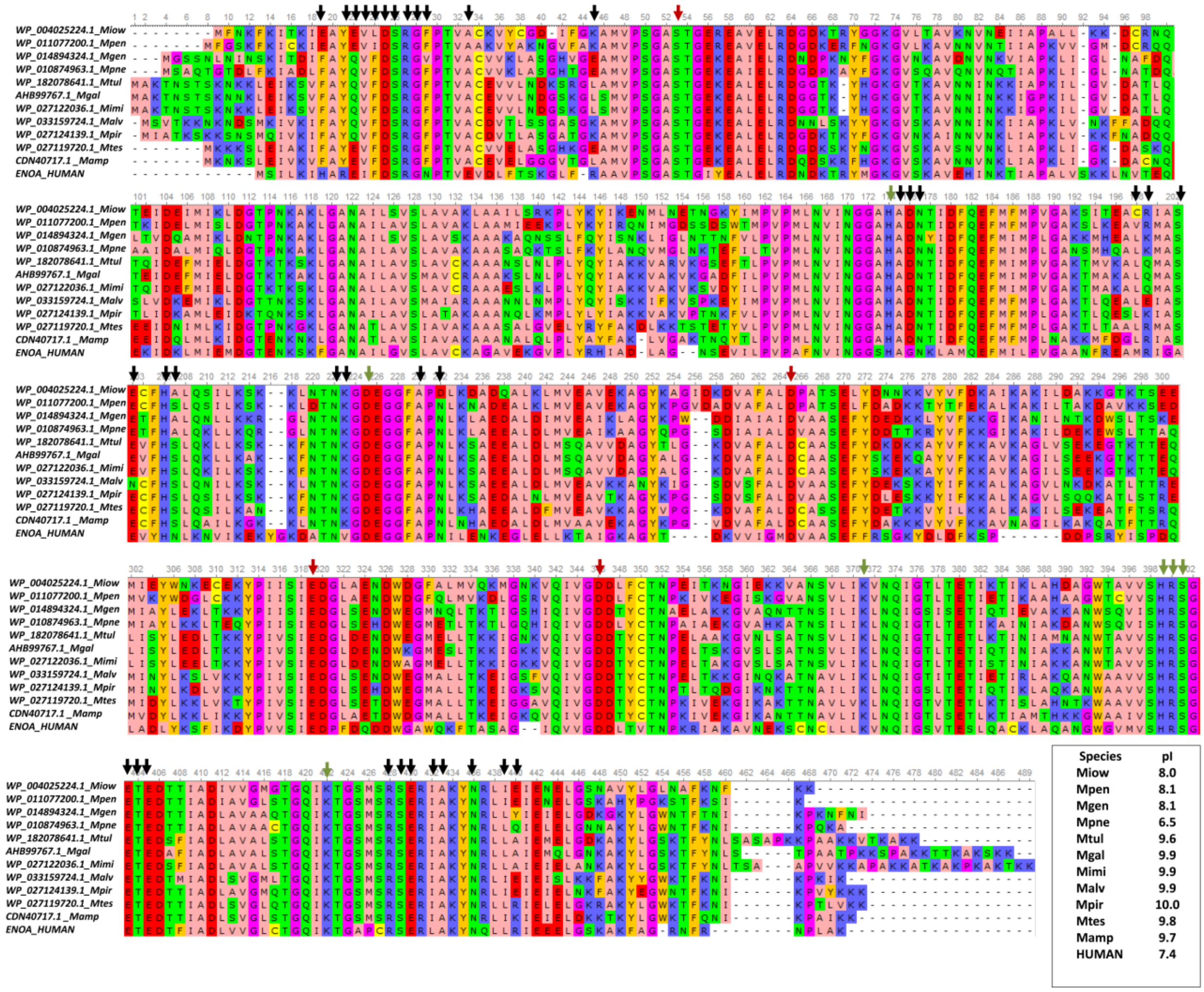

Supplementary figure 9. Multiple protein sequence alignment of enolase orthologs from mycoplasmas and human alfa-enolase. Black, green and red arrows indicate amino acids that form dimer interface, substrate-binding pocket and metal-binding site respectively. The residues are coloured according to their physicochemical properties (Zappo color code, blue for positively charged residues). Miow - *Mycoplasma iowae*; Mpen - *Mycoplasma penetrans*; Mgen - *Mycoplasma genitalium*; Mpne - *Mycoplasma pneumoniae*; Mtul - *Mycoplasma tullyi*; Mgal - *Mycoplasma gallisepticum*; Mimi - *Mycoplasma imitans*; Malv - *Mycoplasma alvi*; Mpir - *Mycoplasma pirum*; Mtes - *Mycoplasma testudinis*; Mamp - *Mycoplasma amphoriforme*. The panel in the bottom-right corner shows the isoelectric point values for the respective enolases.
